# Nutrition management of PKU with pegvaliase therapy: update of the web-based PKU nutrition management guideline recommendations

**DOI:** 10.1186/s13023-023-02751-0

**Published:** 2023-06-22

**Authors:** Amy Cunningham, Fran Rohr, Patricia Splett, Shideh Mofidi, Heather Bausell, Adrya Stembridge, Aileen Kenneson, Rani H. Singh

**Affiliations:** 1 Hayward Genetics Center, Tulane University School of Medicine, 1430 Tulane Ave SL-31, New Orleans, LA USA; 2 Met Ed Co, Boulder, CO USA; 3 Splett and Associates, Stanchfield, MN USA; 4 Maria Fareri Children’s Hospital/Westchester, New York Medical College, Hawthorne, NY USA; 5 Ann and Robert H. Lurie Children’s Hospital of Chicago, Chicago, IL USA; 6 Department of Human Genetics, Emory University, Atlanta, GA USA

**Keywords:** Phenylketonuria, PKU, Phenylalanine ammonia lyase, Pegvaliase, Palynziq®, Nutrition guideline, Nutrition

## Abstract

**Background:**

The web-based GMDI/SERN PKU Nutrition Management Guideline, published before approval of pegvaliase pharmacotherapy, offers guidance for nutrition management of individuals with phenylketonuria (PKU) treated with dietary therapy and/or sapropterin. An update of this guideline aims to provide recommendations that improve clinical outcomes and promote consistency and best practice in the nutrition management of individuals with PKU receiving pegvaliase therapy. Methodology includes: formulation of a research question; review, critical appraisal, and abstraction of peer-reviewed studies and unpublished practice literature; expert input through Delphi surveys and a Nominal Group process; and external review by metabolic experts.

**Results:**

Recommendations, summary statements, and strength of evidence are included for each of the following topics: (1) initiating a pegvaliase response trial, (2) monitoring therapy response and nutritional status, (3) managing pegvaliase treatment after response to therapy, (4) education and support for optimal nutrition with pegvaliase therapy, and (5) pegvaliase therapy during pregnancy, lactation, and adolescence. Findings, supported by evidence and consensus, provide guidance for nutrition management of individuals receiving pegvaliase therapy for PKU. Recommendations focus on nutrition management by clinicians, as well as the challenges for individuals with PKU as a result of therapy changes.

**Conclusions:**

Successful pegvaliase therapy allows the possibility for individuals with PKU to consume an unrestricted diet while still maintaining the benefits of blood phenylalanine control. This necessitates a perspective change in education and support provided to individuals in order to achieve healthy nutrient intake that supports optimal nutritional status. The updated guideline, and companion Toolkit for practical implementation of recommendations, is web-based, allowing for utilization by health care providers, researchers, and collaborators who advocate and care for individuals with PKU. These guidelines are meant to be followed always taking into account the provider’s clinical judgement and considering the individual’s specific circumstances. Open access is available at the Genetic Metabolic Dietitians International (https://GMDI.org) and Southeast Regional Genetics Network (https://managementguidelines.net) websites.

## Introduction

Phenylketonuria (PKU) (OMIM **#** 261,600) is an inherited metabolic disorder (IMD) resulting from gene mutations that impair the function of the enzyme phenylalanine hydroxylase (PAH) [1]. If not diagnosed and treated from infancy, PAH deficiency causes neurotoxic elevations of blood phenylalanine (PHE) concentrations that can result in developmental delay and irreversible neurologic damage. The goal of PKU therapy is to maintain blood PHE concentrations within a therapeutic range, while providing optimal nutrition to support normal growth and development [2]. Historically, this has been accomplished by restriction of dietary PHE to that required for anabolism, supported by intake of medical food formulas, and specialty low-protein food products [3]. This approach to therapy broadened with the approval of the first adjunct pharmacotherapy, sapropterin dihydrochloride, in 2007 [4].

A web-based PKU Nutrition Management Guideline was published in 2015 as part of a multi-year project with the collaboration of Genetic Metabolic Dietitians International (GMDI) and Southeast Regional Genetics Network (SERN) [2]. The goal of this ongoing project is to develop nutrition management guidelines for rare IMDs that foster optimal and consistent guidance for nutrition management of affected individuals. Companion Toolkits for each disease state provide practical guidance for clinicians in implementing guideline recommendations. The guideline can be openly accessed from the GMDI (https://GMDI.org) and SERN (https://managementguidelines.net) websites.

Pegvaliase (Palynziq® BioMarin, Novato, CA) was approved for use by the United States (US) Food and Drug Administration (FDA) in 2018 and by the European Medicines Agency (EMA) in 2019 as an alternate pharmacotherapy for management of PKU and its use in metabolic centers is rapidly expanding [5, 6]. Pegvaliase is an injectable form of phenylalanine ammonia lyase (PAL), an enzyme that metabolizes blood PHE, and substitutes for compromised PAH enzyme activity [7]. The goal of pegvaliase therapy for an individual with PKU is to achieve blood PHE control while consuming a healthy diet containing adequate protein, and without the need for medical food [8]. Successful pegvaliase therapy can provide treatment benefits, optimize nutritional status, and improve quality of life (QoL). Because pegvaliase therapy may allow a diet unrestricted in dietary protein, nutrition management must shift toward supporting healthy nutrition and dietary choices, without PHE restriction. Refocused education and counseling for individuals whose successful treatment was previously dependent on controlled dietary therapy becomes essential.

With the expanding adoption of pegvaliase in clinical practice, a systematic review of literature and practice related to nutrition management with pegvaliase therapy was undertaken to develop care recommendations. The primary research question for the update to the PKU Nutrition Management Guideline and companion Toolkit was, *For individuals with PKU whose therapy includes pegvaliase as a treatment option, what medical and nutritional considerations are associated with positive outcomes?* The five areas of focus for developing recommendations in support of this question were: (1) initiating a pegvaliase response trial, (2) monitoring therapy response and nutritional status, (3) managing pegvaliase treatment after response to therapy, (4) education and support for optimal nutrition status with pegvaliase therapy, and (5) pegvaliase use during pregnancy, lactation, and adolescence*.* In 2022, the web-based PKU Nutrition Management Guideline and companion Toolkit were updated to incorporate this new guidance related to pegvaliase use.

This report describes the process used to synthesize evience from published sources and clinical experts, and summarizes the resultant updated GMDI/SERN PKU Nutrition Management Guideline recommendations, including nutrition management of individuals receiving pegvaliase therapy.

Because nutritional management for pegvaliase therapy must be closely aligned with medical management, the cooperative and knowledgeable expertise of physicians, nurse practitioners (NP), physicians assistants (PA), and dietitians experienced in treatment of PKU is essential. An overview of medical management is addressed in the guideline, as it is important for each member of a clinical team to understand all aspects of treatment.

## Methods

The methodology used in conducting the search for evidence, its critical appraisal and synthesis, and resulting clinical recommendations for the guideline update followed the same rigorous, transparent, and systematic process [9] described for development of the original PKU Nutrition Management Guideline [2] and three other previously published nutrition management guidelines [10–12]. This methodology is consistent with widely accepted standards for systematic review and guideline development [13], with the addition of explicit consensus techniques (Delphi surveys [14] and Nominal Group meetings [15]) to incorporate professional experience and expertise where research is lacking for this rare disease state. The process was implemented by the PKU Pegvaliase Workgroup under the direction of the Nutrition Guideline Project Core Team. These groups are comprised of registered dietitian nutritionists (RDNs) with extensive experience managing metabolic disease therapy as members of multidisciplinary teams in metabolic centers across the US. Each step of the process is fully and transparently documented and archived on the SERN portal (https://managementguidelines.net).

The guideline development process included a systematic review, recommendation development and verification, and document preparation as follows.

1. Key topics were identified and the PICO approach, from evidence-based medicine (**P**atient/problem/population, **I**ntervention, **C**ontrol/comparison, **O**utcome), was used to formulate research topics and literature search strategies.
2. A search for published (formal) research as well as clinical protocols and unpublished studies (gray literature), was followed by adjudication to verify relevance. PubMed was utlilized for the literature search conducted on 30 November 2021, using key words mapped to MeSH (Medical Subject Heading) terms by a specialist librarian. Inclusion criteria were: human studies published during or after January 2015 in the English language. Exclusion criteria were: studies unrelated to pegvaliase treatment or outcome, animal studies, and general nutrition and genetics overview articles. The search for gray literature involved a call for documents using the GNO-METAB- L listserv, a global resource tool facilitating communication among genetic metabolic dietitians, and direct contact with US clinics having known experience with pegvaliase therapy, as well as review of conference proceedings.
3. Critical appraisal of formal and gray literature was conducted by trained analysts using Quality Criteria Checklists (available from authors upon request).
4. Abstraction of pertinent information to Overview Tables (study sample, intervention or factors of interest, findings, limitations, and contribution to the preselected topics) was completed by trained workgroup members.
5. A Delphi survey (Delphi 1) of experts with pegvaliase experience (4 medical doctors, 4 nurse practitioners, and 17 metabolic dietitians) focused on clinical practices not addressed in the literature and areas of uncertainty or variation in practice. Anonymous responses indicated level of agreement with clinical practice statements using a 7-point Likert scale, along with comments. Consensus was defined as ≥ 80% agreement with the clinical statement.
6. Literature and Delphi 1 findings were synthesized into preliminary recommendations and evidence summaries for each topic, with source of evidence (research literature or consensus-based) clearly identified.
7. A virtual Nominal Group meeting was convened to discuss clinical issues unresolved from the literature review or Delphi 1 responses. Nominal Group participants included 10 invited medical and nutrition experts active in pegvaliase research and clinical care, individuals having personal experience with PKU or as a PKU caregiver, and a representative of the National PKU Alliance. Nominal Group members did not include Delphi respondents. All received prelimary recommendations and evidence summaries prior to the meeting.
8. Incorporating responses from the Nominal Group, a second Delphi survey (Delphi 2), focusing on remaining areas of uncertainty or variation in practice was then conducted with the previous Delphi 1 participants.
9. Consensus-based evidence was integrated with that from formal and gray literature to finalize recommendations and evidence summaries and to prepare concise conclusion statements.
10. Strength of evidence (insufficient evidence, consensus, weak, fair, or strong) was rated for each recommendation. For definitions of strength of evidence terms see the web-based PKU Nutrition Management Guideline Appendix A (https://GMDI.org and https://managementguidelines.net).
11. All substantive information was reviewed by the Core Team to finalize recommendations for pegvaliase therapy for the PKU Nutrition Management Guideline update.
12. An external review by experts was conducted.

A list of finalized guideline questions and topics developed through the process described can be found in Table 1.

**Table 1:** Recommendations for individuals receiving pegvaliase therapy and strength of recommendations

Recommendations	Strength of evidence
1. Initiating a pegvaliase response trial	
1.1 Include a metabolic dietitian and a metabolic physician or nurse practitioner, at a minimum, on the clinical team to manage individuals with PKU on pegvaliase therapy. Also consider including a psychologist, social worker, nurse, diet technician and clinic coordinator	Consensus
1.2 Consider offering pegvaliase as a treatment option for adults with blood PHE > 600 µmol/L (including women who are not planning a pregnancy in the next year), adults on sapropterin therapy, adults with blood PHE < 600 µmol/L who want to achieve a normalized or unrestricted diet, and adults with neurocognitive deficits (including late-treated individuals; see also special populations)	Fair
1.3 For individuals considering pegvaliase therapy, review the possibility of adverse events (AE) during treatment, as well as AE management, including Risk Evaluation and Mitigation Strategies (REMS)	Strong
1.4. Discuss the clinic’s expectations for the individual initiating therapy including: the clinic’s protocol (visits, blood monitoring, communication, reasons for discontinuation) and typical course (time to response, dose and number of injections, and diet adjustments)	Fair
1.5 In coordination with the medical team, consider pre-medications (including H1 and H2 receptor antagonists) prior to injections and/or with dose increases to manage or prevent adverse events	Fair
1.6 Initiate and titrate pegvaliase injections as described in the prescribing information and make dose adjustments based on clinical judgement of individual tolerance and blood PHE response	Strong
1.7 Provide weekly individual-clinic contact (via phone, email, in-person or telehealth) to assess therapy tolerance and provide support. Monitor medical and nutrition progress every 2–4 months during therapy initiation and titration	Consensus
2. Recommendations for assessing and monitoring nutritional status during all phases of pegvaliase therapy	
2.1 Establish at the clinic level standard repeatable methods for nutrition assessment and for assessing food attitudes, aversions, and fears related to normalizing a PHE-restricted diet. Conduct a comprehensive nutrition assessment at baseline, including dietary intake and typical eating patterns (using diet records, 24-h recall, food frequency questionnaires), with particular attention to protein adequacy [quantity, quality, and source of protein (plant, animal, synthetic)], and transition from medical food. Continue to evaluate the nutritional adequacy of dietary intake throughout treatment and recommend modifications and/or supplementation as needed	Fair
2.2 Assess the individual’s dietary goals, food behavior and beliefs, and knowledge of what constitutes an unrestricted healthy diet. Include assessment of taste or texture aversions, fears and phobias of previously forbidden foods, and preferences for new foods to include as the diet is normalized	Weak
2.3 Establish at the clinic level standard repeatable methods for assessing quality of life and neurocognitive assessment	Weak
2.4 Monitor blood PHE and TYR concentrations every 1–4 weeks during initiation of pegvaliase therapy. Monitor with increased frequency during dose titration (some individuals may respond before the recommended titration schedule is completed) and diet adjustment periods, and during periods of hypoPHE (< 30 µmol/L)	Fair
2.5 Monitor nutritional status biomarkers including: plasma amino acids, prealbumin, ferritin, vitamin D, and CBC at baseline and every 6–12 months (as recommended in the GMDI Nutrition Management for PKU Guideline). If dietary intake is suboptimal, or other indications of poor nutritional status are present, consider monitoring additional biomarkers including but not limited to vitamin B12, MMA, RBC folate, EFA, Fe, Zn, Se, lipid profile and Hgb A1c	Weak
2.6 Review information pertinent to nutritional status in the medical history and physical exam. Assess physical signs of nutritional status at baseline and throughout therapy. Monitor vital signs and AEs weekly until week 24 and monthly thereafter with attention to vital signs prior to injection and for one hour post injection	Fair
3. Recommendations for managing pegvaliase therapy after a response	
3.1 Aim for a blood PHE concentration below 360 µmol/L and above 30 µmol/L	Fair
3.2 Consider any clinically meaningful reduction in blood PHE concentration (as determined by the clinic) to be a positive outcome	Consensus
3.3 For individuals receiving sapropterin therapy, continue sapropterin until blood PHE is in the treatment range (as defined by the clinic) and then discontinue	Fair
3.4 Add protein in 10–20 g increments when blood PHE concentration falls to 120 μmol/L on two or more consecutive measurements and consider adding protein earlier if blood PHE levels are dropping rapidly. Continue adding intact protein incrementally until the DRI (0.8 g/kg for adults) is met or exceeded (by up to 1.5–2.0 times DRI), considering individual needs and preferences	Fair
3.5 If blood PHE concentrations fall to < 30 µmol/L on two or more consecutive measurements, increase intact protein to meet the DRI or greater. If blood PHE remains below 30 µmol/L after increasing protein intake to at least the DRI, decrease total weekly pegvaliase dose	Fair
3.6 Aim to eliminate medical food by decreasing incrementally as intact protein is increased	Fair
3.7 If post-prandial blood TYR concentrations repeatedly fall below 30 µmol/L, consider increasing intact protein or providing TYR supplementation	Weak
3.8 Aim for a positive therapy outcome that recognizes an individual’s preferences and goals. The goal of pegvaliase therapy is to maintain blood PHE control while consuming an unrestricted healthy diet with adequate intact protein and no medical food. A positive outcome is also achieved when individual preferences for degree of diet normalization, some continuation of medical food, and/or number of dose injections required for blood PHE control are taken into account	Weak
4. Recommendations for educating and counseling regarding appropriate food choices to support normal nutrition requirements	
4.1 Identify and address individual expectations and goals regarding benefits of pegvaliase therapy including normalization (containing at least DRI for protein and no medical food) or liberalization of diet while maintaining blood PHE control; and improving quality of life psychosocial well-being, and cognitive function. Continue to address expectations and goals as they change throughout therapy	Fair
4.2 Provide ongoing education and counseling for transitioning to an unrestricted healthy diet including: addition of high protein foods, appropriate portion sizes, food safety, food purchasing and preparation, and weight management. Provide ongoing education and counseling for transitioning to an unrestricted diet	Weak
4.3 Educate individuals who follow a predominantly vegetarian diet on how to incorporate adequate protein and micronutrients, including micronutrient supplements if necessary	Fair
4.4 Recognize and provide counseling for individuals who have difficulty accepting foods that were previously not allowed, and refer to a psychologist or other professional, if indicated	Weak
5. Recommendations for pegvaliase use in special populations	
5.1 Counsel women of childbearing age about the limited information available on pegvaliase in pregnancy and the need for birth control to prevent unexpected pregnancies	Consensus
5.2 For women receiving pegvaliase therapy, consider continuing pegvaliase during pregnancy, weighing the risks of the known teratogenic effects of high blood PHE on the fetus with the unknown risks of pegvaliase, and incorporating the woman's preferences	Consensus
5.3 For women continuing pegvaliase therapy during pregnancy, aim for blood PHE concentration between 120 and 360 µmol/L and monitor blood PHE weekly to bi-weekly	Weak
5.4 For women receiving pegvaliase who return to a PHE-restricted diet for pregnancy, discontinue pegvaliase at least 4 weeks prior to a planned pregnancy	Fair
5.5 For women who are returning to a PHE-restricted diet for pregnancy, provide frequent nutrition counseling and support	Weak
5.6 Consider pegvaliase therapy during lactation with frequent monitoring of maternal blood PHE	Consensus
5.7 Consider pegvaliase treatment for individuals aged 16 years and older, taking into consideration level of blood PHE control, neurocognitive status, and quality of life on current therapy, as well as the individual's interest	Weak

## Results

A literature search resulted in 24 published original research articles and 12 reviews (for a total of 36 formal literature sources), 4 unpublished poster presentations, and 20 protocols or other materials used in clinical practice (for a total of 24 gray literature sources) that met criteria for evidence synthesis. Consensus for resolving practice issues not adequately addressed in those sources were systematically explored through Delphi surveys and a Nominal group meeting. Results are detailed on the GMDI/SERN portal (https://managementguidelines.net). Recommendations for each topic and evidence from which they were derived follows.

**1. Summary of evidence: Initiating a pegvaliase response trial** (Table 1, Recommendation 1).

The following recommendations describe the need for a multidisciplinary metabolic team and considerations for initiating appropriate individuals on pegvaliase therapy.

1.1 Individuals starting pegvaliase therapy are best managed by a multidisciplinary team, including a metabolic dietitian and a metabolic physician or nurse practitioner, at a minimum.

The multidisciplinary team should consider including a psychologist, social worker, nurse, diet technician, and clinic coordinator. In their respective publications, Burlina and Rocha recommended including a nurse practitioner or a nurse, and a psychiatrist or psychologist for evaluation of neurological health [16, 17]. Two metabolic centers have reported their multidisciplinary teams include metabolic geneticists, a nurse or nurse practitioner, and metabolic dietitians. One clinic also included a psychologist [18, 19]. Management of individualized pegvaliase therapy may be complicated, and is best provided only by a team of experienced clinicians.

1.2 Identify appropriate individuals for a pegvaliase trial. Consider adults with blood PHE > 600 µmol/L (including women who are not planning a pregnancy in the next year), adults on sapropterin therapy, adults with blood PHE < 600 µmol/L who desire an unrestricted diet, and adults with neurocognitive deficits (including late-treated individuals).

Pegvaliase is approved in the US for adults with PKU who have blood PHE concentrations > 600 µmol/L and who have not been able to achieve good metabolic control with previous therapies. Published guidance also suggests considering pegvaliase for adults with good therapy adherence [8], recognizing that a blood PHE of 600 µmol/L is above the recommended treatment goal of < 360 µmol/L and that individuals who follow treatment recommendations should also have the option of maintaining blood PHE control while consuming an unrestricted or normalized diet without the need for medical food.

Individuals with high blood PHE have neurocognitive deficits that may interfere with their ability to follow a pegvaliase treatment regimen. However, pegvaliase should be considered for all adults with PKU as long as they are able to give informed consent to treatment and adhere to treatment recommendations, including the requirements for monitoring adverse events (AE) [8]. Recommendations are to consider pegvaliase therapy to achieve life-long maintenance of blood PHE close to normal concentrations while normalizing intact protein intake [16, 17]. There was consensus among Delphi 2 survey respondents that late-treated individuals with PKU may be considered for pegvaliase therapy. Comments included that the individual must be able to recognize and verbalize AEs, including signs and symptoms of anaphylaxis, and be able to use an auto-injector epinephrine pen (EpiPen). They should also be supervised by care partners prepared to respond in case of anaphylactic reaction. (Recommendations for the use of pegvaliase in adolescence, pregnancy and lactation are found in Table 1, Recommendation 5).

1.3 Prepare the individual for a pegvaliase trial and discuss the risks associated with treatment and how risks will be mitigated.

The most commonly reported AEs associated with pegvaliase treatment include arthralgia, injection site reactions, and headaches [8]. There also is a possibility of anaphylaxis. The metabolic team should discuss the manufacturer requirements for a prescribed auto-injector epinephrine, and for enrollment in the Risk Evaluation and Mitigation Strategy (REMS) program, an FDA-required drug safety program to ensure the benefits of a medication outweigh its risks. Adverse events associated with pegvaliase are generally well-tolerated but can be significant and lead to discontinuation of the medication. In one clinic's real-world experience, 8 of 37 patients (21.6%) discontinued pegvaliase, with two eventually restarting the drug [18]. At a separate clinic, 3 of 21 patients (14%) who started pegvaliase discontinued the drug, but only one of these was due to AEs [19]. Published recommendations state that the decision to discontinue pegvaliase therapy should be at the discretion of the treating clinician and include preference of the individual receiving treatment [8]. Reports vary, but depending on the individual, pegvaliase may either be stopped entirely or titrated with reinstitution of previous dietary treatment. Pegvaliase treatment can be resumed after interruption, with some evidence suggesting that the previous dose can be resumed after interruption of up to 8 weeks. If interruption has been greater than 8 weeks, pegvaliase can be resumed at a lower dose and then escalated at the discretion of the clinician [8].

1.4 Prior to starting pegvaliase, educate individuals on what they can expect from the clinic (dose escalation, time to response, AE monitoring, and support) as well as what the clinic expects from the individual (blood PHE monitoring, attending clinic visits, keeping diet constant unless counseled otherwise, and reporting AEs).

This guidance is consistent with published recommendations [8, 18–20].

Specific clinic protocols containing these common elements should be reviewed. Example protocols are available in the Toolkit that accompanies the web-based GMDI/SERN PKU Nutrition Management Guideline (https://GMDI.org and https://managementguidelines.net).

1.5 Consider pre-medication with H1 and H2 receptor antagonists with initiation of pegvaliase injections, and/or with dose increases to manage or prevent AEs.

The use of pre-medications varies in practice, as reported in both in formal and gray literature. Published medical guidance recommends H1 (a class of medication used to block the action of histamines) and H2 receptor antagonists (a class of medication used to suppress gastric acid secretion), as well as antipyretics, may be prescribed by a physician or other medical provider (eg NP, PA) to prevent or limit common reactions to pegvaliase. They may be given prior to the first injection and daily during the titration phase, then discontinued based on clinical judgement once a stable pegvaliase dose is achieved [8, 17]. However, some clinics prescribe these medications only if an AE occurs. Consensus was reached only for prescribing H2 antagonists prior to dosing.

1.6 Follow FDA approved prescribing information for pegvaliase initiation and titration [21].

A typical induction dose, prescribed by a physician or other medical provider is 2.5 mg weekly for 4 weeks. The dose is then titrated, based on tolerability, over at least 5 weeks to 20 mg/day. If there is no response after 24 weeks, the dose may be increased to 40 mg/day. Formerly, discontinuation of pegvaliase was recommended if there was no response after 16 weeks at 40 mg/day [22], but updated prescribing information is dose may be increased to 60 mg/day and current practice indicates higher doses are used off-label [23].

Effective induction and titration varies by individual. Many individuals require slow titration, especially in cases where AEs are severe or difficult to manage. In some individuals, a response is seen at a dose lower than 20 mg/day, while others may require an increase to 40 mg/day before 24 weeks on the 20 mg/day dose [8]. In one report of 37 individuals starting pegvaliase therapy, 35 were able to follow the standard dosing recommendation and two slowed titration due to AEs [18].

1.7 Additional clinic support is recommended for the individual starting pegvaliase.

There was consensus that communication between the individual and the metabolic team should occur weekly via phone, email, in-person, or telehealth and should both provide support and assess therapy tolerance. Guidance should be given to maintain dietary management until a response with associated lowering of blood PHE is achieved. Close monitoring of medical and nutrition progress every 2–4 months during therapy initiation and titration also is recommended. However, clinic support may vary from weekly to quarterly, with more visits needed during the titration phase [18]. Visit frequency may also depend on distance from the clinic, access to telehealth, and availability of clinic personnel.

Of the pegvaliase clinic protocols examined, most suggested a medical and/or nutrition visit every month, with weekly check-ins with the clinical team. In addition, many clinics work with industry-based Clinic Coordinators who offer home or telehealth visits weekly during titration [18]. These visits reinforce injection training and often continue until an individual is comfortable injecting, at each dose increase, and as needed for support and education.

**2. Summary of Evidence: Assessing and monitoring nutritional status during all phases of pegvaliase therapy** (Table 1, Recommendation 2).

Nutrition monitoring is a key element of appropriate management for all individuals with PKU and monitoring the clinical and nutritional status of an individual with PKU on pegvaliase therapy is especially necessary for those in transition to an unrestricted diet. The inherent limitations of the PKU diet can result in increased risk for nutritional insufficiencies. Assessment of dietary intake (specifically protein), nutrient analysis, and evaluation of clinical signs and symptoms of nutritional insufficiencies in addition to biochemical monitoring of PHE, tyrosine (TYR), and markers of at-risk nutrients are critical to the successful unrestriction or normalization of the diet of an individual on pegvaliase therapy. See GMDI/SERN PKU Nutrition Management Guideline for nutritional status monitoring recommendations in individuals with PKU (https://managementguidelines.net) [2].

Recommendations for assessment and monitoring during pegvaliase therapy are outlined below.

2.1 Conduct a comprehensive nutrition assessment at baseline and repeat assessments during diet adjustment and maintenance phases by evaluating dietary intake and typical eating patterns (eg, diet records, 24 h recall, food frequency questionnaires).

A European consensus report proposed assessment of nutritional intake using food frequency questionnaires (FFQ), 24-h recall, or 3-day food records, up to once per month during the titration and dosing optimization phase and every 6 months during the maintenance and diet normalization phase [17]. In one post-clinical trial study, dietary assessment with a 3-day food record and FFQ estimated the nutritional quality of the diet of individuals who responded to pegvaliase [24]. Clinic protocols reviewed emphasized evaluating current dietary intake using diet records, FFQ, or typical eating patterns of the individual (including medical food used) during the initiation visit to assess adequacy of the diet and during every adjustment based on dose/PHE concentrations.

Because only a few studies evaluating use of pegvaliase have been published since the clinical trials, recommendations are based primarily on consensus methodology. Consensus agreement (100%) was reached for evaluating dietary intake at baseline and with each diet adjustment, including protein quantity, quality, and source (plant, animal, synthetic), transition from medical food, and food variety and acceptance. There was consensus that use of food records to track protein intake is needed for individuals both on or off a PHE-restricted diet. The Delphi 1 survey showed 100% overall agreement to evaluate individual food behaviors and attitudes. Registered dietitians specifically noted this was critical to their counseling, with 60% agreement to specifically look at food neophobia.

2.2 Nutrition assessment should include the individual’s dietary goals, food behavior and beliefs, and knowledge of what constitutes an unrestricted healthy diet.

Since patients treated with pegvaliase may transition from a lifelong signficantly restricted diet to an unrestricted diet over the course of treatment, it is recommended that assessment include discussion about taste or texture aversions, fears and phobias of previously forbidden foods, and preferences for new foods to include as the diet is normalized. There was widespread agreement in the Delphi 2 survey for assessing food behaviors and beliefs, as well as recognition of the need for tools to explore methods for assessing and documenting food attitudes, aversions, and fears related to liberalizing/normalizing a PHE-restricted diet.

2.3 Establish at the clinic level standard repeatable methods for assessing quality of life and neurocognitive assessment.

Improvements in neurocognition and neuropsychological outcomes, QoL, and emotional well-being of individuals with PKU are treatment goals of pegvaliase therapy. One European review recommends full IQ assessment, with neurocognition and psychiatric follow-up tailored to an individual’s clinical and life conditions [17]. Validated assessment tools for self-report of QoL, neurocognition, and neuropsychological issues specific to individuals with PKU are available [25, 26], and are recommended as part of clinical follow-up and overall PKU management [3, 16]. While QoL impacts therapy adherence and social and neurocognitive profiles, published systematic research related to these outcomes is limited.

2.4 Monitor blood PHE and TYR concentrations every 1–4 weeks during initiation of pegvaliase therapy, with increased frequency during dose titration (some individuals may respond before the recommended titration schedule is completed), and diet adjustment periods, and during periods of hypophenylalaninemia (hypoPHE) when blood PHE is < 30 µmol/L.

Blood PHE and TYR measurements were frequently monitored during the pegvaliase clinical trials [8, 27]. During post-marketing studies, blood PHE and TYR were monitored every 2 weeks at one metabolic clinic [18] and every month at another clinic, until the diet was changed, at which time monitoring frequency was increased to weekly [19].

2.5 Other biomarkers of nutritional status that should be monitored include, plasma amino acids, prealbumin, ferritin, 25-hydroxy vitamin D, and complete blood count (CBC) at baseline and every 6–12 months. If dietary intake is suboptimal, additional biomarkers to consider are vitamin B12, methylmalonic acid (MMA), red blood cell count (RBC), folate, essential fatty acids (EFA), iron (Fe), zinc (Zn), selenium (Se), lipid profile, and hemoglobin A1c (Hgb A1c).

These nutritional status markers and frequency of monitoring are based on the GMDI/SERN PKU Nutrition Management Guideline monitoring table (https://managementguidelines.net) [2] with the note that decisions must be based on individual needs and clinical judgement.

A cross-sectional study of the nutritional status of 18 individuals prior to initiating pegvaliase therapy examined vitamin and mineral status with laboratory monitoring of vitamins A, E, and 25-hydroxy vitamin D, RBC, folate, serum Fe, ferritin, transferrin saturation, magnesium (Mg), MMA, Se, and Zn. Generally, individuals studied had normal nutritional status, but some exhibited low 25-hydroxy vitamin D (< 30 ng/mL) and/or low ferritin [19]. The same individuals were evaluated for nutritional status after treatment with pegvaliase was established, with markers for protein and EFA status, lipid profile, Hgb A1c, vitamins A and E, 25-hydroxy vitamin D, RBC, folate, Fe, Mg, Se, Zn, and MMA, all reported as normal [24].

Guidance in Europe, where experience is more limited, recommends, at a minimum, assessing anthropometrics and food intake. In addition, plasma amino acids, prealbumin, CBC, ferritin, total homocysteine or MMA, folic acid, vitamin B12, 25-hydroxy vitamin D, Fe, calcium (Ca), Zn, and Se are strongly recommended. Body composition (fat free body mass, phase angle obtained from bioelectrical impedance analysis) and bone mineral density (DXA scan) are also recommended [17].

Supplementation of vitamins, minerals, and other micronutrients should be recommended when insufficiency is noted. Use of a multivitamin and/or individual Ca, Fe, vitamin B12, and biotin supplements for individuals with inadequate nutrient intake should be considered [8].

2.6 Nutrition and medical providers should review information pertinent to nutritional status in the medical history and physical exam, assess physical signs of nutritional status at baseline and throughout therapy, and monitor vital signs and AEs weekly until week 24 and monthly thereafter, with attention to vital signs prior to injection and for one hour post injection.

Evidence- and consensus-based recommendations indicate that individuals should be assessed (in person or by telephone/telemedicine) for signs and symptoms of hypersensitivity reactions every 2–4 weeks during introduction and initial titration of pegvaliase and during dose changes [8]. Vital signs and safety measures should be evaluated at screening, then weekly, and then every 4 weeks thereafter when response is noted [27]. Clinic protocols included monitoring vital signs prior to injection and one-hour post injection. Anthropometric evaluation including weight, height, body mass index (BMI), and waist circumference was proposed as a recommendation for minimal nutritional status assessments at up to once per month during the titration and dosing optimization phase and every six months during the maintenance and diet normalization phase [17].

Attention to nutrition-related physical assessment was raised in a study evaluating nutrition status of pegvaliase responders. A nutrition focused questionnaire revealed 5 of 11 females experienced hair loss before and during treatment. The researchers were not able to correlate this finding with other clinical factors such as blood PHE, protein intake, or pegvaliase dosing [19].

**3. Summary of evidence: Recommendations for managing pegvaliase therapy after a response** (Table 1, Recommendation 3).

The third set of recommendations refer to managing pegvaliase therapy after response, including blood PHE and TYR monitoring, pegvaliase dosage adjustment, modification of dietary protein and medical food intake, and supplementation of deficient nutrients.

3.1 Aim for a blood PHE concentration of 30–360 µmol/L.

American College of Medical Genetics (ACMG) guidelines recommend an optimal blood PHE concentration of ≤ 360 µmol/L [28]. In pegvaliase clinical trials, 44% of participants achieved blood PHE concentrations of ≤ 360 μmol/L by 12 months and 61% by 24 months. A total of 51% of participants achieved blood PHE levels ≤ 120 μmol/L by 24 months [29].

One article suggested that improvements in specific neurotransmitters may be more likely in individuals who achieve blood PHE concentrations of < 360 µmol/L as compared with individuals with PHE concentrations > 900 µmol/L. The report stated that blood PHE concentrations between 31 and 120 µmol/L should not be regarded as too low [30]. Gray literature included a poster presented by Thomas et al., reporting data from the pegvaliase clinical trials, which addressed outcomes of individuals who developed hypoPHE, as defined by two or more blood PHE results < 30 µmol/L. HypoPHE was observed at all pegvaliase doses evaluated, and appeared to be well tolerated, manageable, and allowed for greater dietary intake of intact protein and PHE. Individuals exhibiting hypoPHE tended to respond to pegvaliase earlier, discontinued the study less often, and did not have more frequent AEs, except for a higher incidence of alopecia [31].

Hair loss and/or skin abnormalities were observed in some individuals with hypoPHE, but there was disagreement as to whether this was caused by the hypoPHE. Recommendations included, as a precaution, that extended periods of hypoPHE (< 30 µmol/L for > 3 months) should be avoided and addressed with diet or pegvaliase dosage adjustments [8].

In the Delphi 2 survey there was 94% agreement that an appropriate target blood PHE concentration for individuals treated with pegvaliase should be < 360 µmol/L and > 30 µmol/L.

3.2 Consider any clinically meaningful reduction in blood PHE concentration (as determined by the clinic) to be a positive treatment outcome.

The efficacy and benefit of pegvaliase therapy should primarily be determined by a significant reduction in blood PHE concentration from baseline, or maintenance of blood PHE concentration within an acceptable range, and with progress in normalizing diet, which was defined as ability to consume more intact protein with reduced requirement for medical foods [8]. Clinically meaningful efficacy should be determined by the clinician and also be based on an individual’s goals [8]. Additional benefits to consider when determining efficacy include a reduction in disease burden, an improvement in QoL, and an increase in psychosocial well-being and/or cognitive function [8, 17].

3.3 Individuals who are concurrently receiving sapropterin therapy should continue sapropterin until blood PHE is in the treatment range and then discontinue it.

Published recommendations state that the decision to discontinue sapropterin therapy and administer pegvaliase should be at the discretion of the treating clinician and include individual preference [8].

There is little published data about the use of concurrent pegvaliase and sapropterin therapy. One study reported it is unclear whether concurrent use is more beneficial than monotherapy [22]. One clinic reported 54% (20/37) of individuals who started pegvaliase had concomitant sapropterin use. One individual was denied insurance coverage when using both sapropterin and pegvaliase, and several reported not taking sapropterin after starting pegvaliase injections; yet, the clinic recommended that individuals stay on sapropterin until they respond to pegvaliase (blood PHE < 120 µmol/L on two occasions) [18]. In the Delphi 2 survey, there was consensus that individuals should remain on sapropterin therapy until blood PHE reaches the target range (as defined by the clinic) and no consensus that sapropterin should be continued until the diet is normalized.

3.4 Adjust the diet once there is a response to pegvaliase. For individuals who consume medical food, add intact protein in 10–20 g increments when blood PHE concentration falls to 120 μmol/L on two or more consecutive measurements and consider adding protein earlier if blood PHE levels drop rapidly. Continue adding intact protein incrementally until the DRI (0.8 g/kg for adults) is met or exceeded (by up to 1.5–2.0 times DRI), considering individual needs and preferences.

This guidance is consistent with published recommendations [8]. Protein intake for adults in the US is often higher than the DRI. The 2001–2014 National Health and Nutrition Examination Survey (NHANES) reported adults in the US averaged a higher daily consumption of protein than the DRI (0.8 g/kg ideal body weight/day), ranging from 1.15 g protein/kg ideal body weight to 1.45 g protein/kg ideal body weight [32]. The Acceptable Macronutrient Distribution Range for protein is 10–35% of total energy intake [32] and the range of protein intake reflected in the USDA My Plate guidance for Americans consuming a healthy eating pattern is 1.48–1.86 g/kg/day [33]. A published roadmap for nutrition management in Europe defines normal protein intake as at least 0.8 g protein/kg/day from a mix of animal and plant sources and without any medical food [17].

A recent study investigating the nutritional status of adults on pegvaliase therapy showed the PKU cohort comparable to US adults in nutritional quality of dietary intake, as described by the NHANES 2015–2016 survey's Healthy Eating Index. However, when looking specifically at protein status, mean intake of intact protein foods (eg, meat, poultry, seafood, eggs, soy, nuts, seeds, and legumes) was significantly lower in this cohort than the US sample. This indicates that adults with PKU on pegvaliase therapy, even though they meet the DRI for amount of protein intake, may still have a lower overall consumption of protein than the US average [24]. In the Delphi 2 survey there was 100% agreement with Recommendation 3.4.

3.5 When blood PHE falls below physiologically normal concentrations (< 30 µmol/L), increase intact protein to meet the DRI or greater, and, if blood PHE remains below 30 µmol/L, decrease total weekly pegvaliase dose.

This guidance comes primarily from published recommendations [8] and is mirrored in the Rocha paper recommendations for nutrition management of individuals receiving pegvaliase [17]. In one post-marketing study, 5 of 13 individuals responding to pegvaliase needed less than daily dosing to maintain blood PHE in the target range, with some needing as low as 20 mg/week [18]. Consensus for this recommendation was reached.

3.6 For individuals with PKU who consume medical food, aim to decrease medical food intake incrementally as intact protein is increased.

This recommendation was also stated in both US and European recommendations for pegvaliase therapy [7, 17]. There was no consensus in either Delphi survey regarding how to adjust medical food intake for an individual on pegvaliase therapy, but there was agreement that the goal is to eliminate medical food incrementally as intake of intact protein meets or exceeds the DRI.

3.7 If post-prandial blood TYR concentrations repeatedly fall below 30 µmol/L, consider increasing intact protein or providing TYR supplementation.

The 2018 US evidence- and consensus-based recommendations suggested maintenance of physiologic TYR blood concentrations is important and TYR supplementation should be considered if post-prandial blood TYR is repeatedly < 30 µmol/L [8]. In pegvaliase clinical trials, all study participants were instructed to prophylactically take supplemental TYR (500 mg tablets three times per day with meals), making reports of hypotyrosenima or a need for TYR supplementation during pegvaliase treatment non-informative [29].

In one study, all 37 individuals on pegvaliase therapy for 12 months had blood TYR concentrations in the normal range (monitored every 2 weeks) and none required supplementation [18].

In the Delphi 1 survey there was overall consensus with Recommendation 3.7.

3.8 After response to pegvaliase, aim for a positive therapy outcome that recognizes the individual’s preferences and goals.

The goal of pegvaliase treatment is to provide life-long maintenance of blood PHE concentrations while “normalizing diet”, which is defined as not requiring medical food and containing at least the DRI for protein. However, there is recognition that an individual’s goals and preferences should be included in the pegvaliase treatment plan [8]. One study reporting outcomes after 1.5 years found 50% of individuals who responded to therapy were able to tolerate a normal protein intake (> 0.8 g/kg/day) with no medical food. Individuals who continued diet restriction tolerated a median 50% increase of intact protein and a 25% decrease in medical food protein [19]. A summary of case studies of individuals receiving pegvaliase reported some individuals wished to liberalize but not totally normalize diet, rather than take multiple injections per day to achieve blood PHE control [20]. A positive outcome may also be achieved when individual preferences for degree of diet normalization, some continuation of medical food, and/or number of dose injections required for blood PHE control are taken into account. There was 94% agreement among Delphi 2 survey responders for this Recommendation 3.8.

**4. Summary of Evidence: Educate and counsel regarding appropriate food choices to support normal nutrition requirements: increased intact protein, healthy energy intake, and micronutrient sufficiency** (Table 1, Recommendation 4).

These recommendations guide metabolic nutritionists and other clinical team members as they counsel and educate patients about pegvaliase therapy.

4.1 Discuss goals and expectations with all individuals with PKU who are starting pegvaliase therapy regardless of their diet therapy status.

Monitoring the impact of pegvaliase on diet, QoL, psychosocial well-being, and cognitive function, and managing individualized patient expectations regarding the benefit of pegvaliase therapy is recommended [8]. Because impaired attention has been determined to be a more common domain of ADHD in early-treated adults with PKU, the relationship between an extended period of blood PHE reduction and sustained improvements in symptoms of inattention was examined in a subgroup of participants of the pegvaliase Phase 3 clinical trials. Findings suggested a direct relationship between improvements in inattention symptoms and metabolic control, with greater symptom improvement associated with greater blood PHE reductions. This was especially robust when blood PHE concentrations fell below 360 µmol/L. The authors conclude that in adults receiving pegvaliase therapy, better blood PHE control, possibly combined with increased dietary protein intake, can improve symptoms of inattention and potentially benefit QoL. The authors also postulated greater benefit when blood PHE levels are maintained within a normal range and the diet is normalized [34].

In a cross-sectional study, individuals who were naïve to pegvaliase reported the minimum benefits they believed therapy should provide to make the possible AEs associated with therapy acceptable. These benefits included lessening disease burden and social challenges, as well as limiting barriers to adherence enough to expect improvement in QoL. Only 28.8% reported satisfaction with their current treatment (treatment modalities included diet restriction, medical food, sapropterin, as well as no therapy), and 84.4% indicated difficulty following a PKU diet. Over 50% reported making careless mistakes, difficulty paying attention, avoiding or delaying tasks requiring thought, being distracted by proximity of noise or action, and misplacing things. Subjects reported barriers to the PKU diet as being burdened with weighing/measuring/estimating protein content of foods (60%), feeling less able to lead a normal social life (49%), and believing the PKU diet prevented sufficient nutrient intake (73%). Non-adherence to therapy during the previous week was reported by 76% of participants. The authors concluded that individuals with PKU were willing to tolerate risk of AEs associated with pegvaliase in exchange for improvement in blood PHE levels that beneficially impacted QoL. In this study, those unwilling to receive pegvaliase cited fear of injections (needles), concern about hypersensitivity reactions, and cost of the therapy [35]. In another study describing clinical experience, individuals receiving pegvaliase therapy reported subjective outcomes that included improvement in QoL, verbal communication, and daily living functioning [19]. These findings, similar to those anticipated by pegvaliase naïve individuals, may validate willingness to endure anticipated AEs to achieve improved outcomes with pegvaliase therapy.

In a summary of case studies of individuals receiving pegvaliase, investigators stated that treatment goals and individual expectations varied and should be clearly defined and re-evaluated throughout the course of therapy as goals may change. Any burden associated with therapy should be monitored. Reported expectations included ability to eat a more normal diet, control of blood PHE concentrations that resulted in more clarity of thinking without a restrictive diet, and decrease or discontinuation of medical food. Clinical providers were described as more effective if they understood the challenges associated with changing life-long dietary habits. Ongoing communication between individuals and providers and guidance from the clinic were considered essential [20].

4.2 Provide ongoing education and counseling for transitioning to an unrestricted healthy diet including addition of high protein foods, appropriate portion sizes, food safety, food purchasing and preparation, and weight management.

The 2018 evidence- and consensus-based recommendations for the use of pegvaliase included education for achieving and maintaining normal nutrition requirements, and individualized guidance for transitioning to a “normalized” diet (defined as the ability to consume at least the DRI for protein and to reduce or discontinue medical food). The authors recommended that the metabolic dietitian evaluate nutrient intake and adequacy, especially quality of protein, and counsel individuals on how to introduce high protein foods into their diet, including correct portioning, food safety, and cooking methods [8].

Disordered or atypical eating behavior is reported to be common in individuals who have always been required to follow a restricted PKU diet, with many reporting guilt when consuming high-protein foods. Change in these dietary restrictions allowed by successful pegvaliase therapy should include recognition that individuals may need help to adjust their emotional response to these previously “forbidden” foods [8].

In a summary of case studies describing pegvaliase therapy, individuals were shown to have limited understanding of what constitutes a healthy diet because they had never been allowed unrestricted food choices, or had discontinued therapy. One case study recommended dietary changes be made slowly and with specific recommendations. This summary stated education for a healthy diet should continue throughout maintenance, with assessment of dietary intake and eating behavior during follow-up clinic visits [20]. One cross-sectional study reported individuals treated with pegvaliase for approximately 5 years were able to consume a normal amount of protein, noting a mean intake of 73.2 gm, with 62% from animal protein [24].

A European consensus publication recommended prevention of onset, or increase, of overweight or obesity, with focus on quality of nutrient intake (especially protein) representing an opportunity to optimize body composition, as this is often unbalanced in individuals treated with PKU diet therapy [17].

The risk for overweight/obesity is increased with unhealthy dietary patterns. In a paper reporting nutritional status of individuals treated with pegvaliase, adults with PKU were more likely to be overweight compared to non-PKU controls. Individuals participating in the pegvaliase clinical trials often consumed more calories and experienced weight gain from baseline. The authors suggested that relaxing of dietary restrictions with pegvaliase therapy can enable a healthier diet but may also allow overindulgence of previously restricted foods [24].

Proactive attention to weight control is recommended. One case study described an individual whose discontinuation of dietary protein restrictions resulted in significant weight gain [20]. After one year on pegvaliase, individuals followed in another clinic experienced weight changes that represented improvements in BMI [19]. However, a study of individuals following an unrestricted diet for over 4 years on pegvaliase maintenance therapy found 50% were overweight and 39% were obese. The authors stated that successful transition to a normal diet requires ongoing education for healthy food choices and cooking methods to achieve a well-balanced diet and healthy weight [18].

Clinic protocols reviewed emphasized the importance of education for healthy food choices begining with initiation of pegvaliase, and continuing after response is established and throughout therapy maintenance. Counseling for meeting intact protein goals and balancing essential nutrients was recommended. Resources for education are provided in the PKU Toolkit (https://GMDI.org and https://managementguidelines.net).

Consensus was reached in the Delphi 1 survey regarding the role for metabolic dietitians after an individual has responded to pegvaliase, including maintaining a relationship with the individual, supporting long-term treatment adherence, providing counseling regarding healthy eating, and monitoring nutritional status and adequacy of protein intake. Survey responders agreed education and support are needed regarding appropriate high protein food choices and portioning, tracking increased protein intake in liberalized (but not normalized) diets, weight management, cooking and recipe help, and budgeting and grocery shopping.

4.3 Ensure individuals with PKU who follow a vegetarian diet consume sufficient protein and other nutrients.

US guidelines for the use of pegvaliase suggest that individuals who follow a predominantly vegetarian diet should be educated on how to incorporate high-quality protein into their diet, and individuals who follow a vegan diet should be educated to consume 10–15% more protein than the DRI due to reduced bioavailability of plant protein. Such individuals are considered at risk for micronutrient deficiencies and should be monitored and educated regarding the use of supplements [8]. A European consensus paper recommended an increase of 20% more protein, or 1 g protein/kg/day, for these vegan individuals [17].

This recommendation was not explored in the Delphi surveys.

4.4 Recognize that individuals may have difficulty accepting foods not previously allowed on a restricted diet. Provide counseling and refer to a psychologist or other professional, if indicated.

Individuals receiving pegvaliase therapy may need counseling to transition to inclusion of unfamiliar higher protein foods in their diet. It is recommended that any emotional response, or neophobia, resulting from the inclusion of previously forbidden foods, causing a possible loss of identity, should be recognized and counseling provided [8].

In one study, the Food Neophobia Scale, adapted for PKU, was administered to 18 individuals receiving pegvaliase therapy for approximately 5 years. Respectively, 47%, 35%, and 28% of individuals reported low, moderate, and high degrees of food neophobia. Those with a low degree of neophobia were less fearful of trying new foods and experienced increased enjoyment of food as assessed by an Epicurean Eating Tendency questionnaire [24].

**5. Summary of evidence: Recommendations for pegvaliase use in adolescents, and in pregnant and lactating women** (Table 1, Recommendation 5).

There is little published evidence on pegvaliase therapy in special populations and recommendations are based largely on case studies and expert consensus.

5.1 Counsel women of childbearing age about the limited information regarding the use of pegvaliase during pregnancy.

Evidence regarding the effect of pegvaliase on pregnancy is limited to animal studies (which were outside the scope of this review) and case reports of women both continuing and discontinuing pegvaliase during pregnancy (see GMDI/SERN PKU Nutrition Management Guideline Recommendation 7.5). The pegvaliase prescribing information states that use in pregnancy may cause fetal harm based on animal studies. In these studies, when pregnant animals were exposed to multiples of maximum recommended dosing based on weight, fetal adverse events were noted. However, when rabbits were given only 3 times the maximum dose of pegvaliase or rats were given less than the maximum dose, no adverse effects on embryo-fetal development were reported [16].

Clinic protocols reviewed call for women with PKU who are of childbearing age and starting pegvaliase therapy to receive counseling (from the RD, genetic counselor, or other clinical team member) about the need for birth control. Some centers require individuals to sign a contract acknowledging the information shared. In addition, several metabolic clinics have pegvaliase protocols that require women with PKU who are considering pregnancy to receive counseling about the risks associated with maternal PKU syndrome (MPKU) and the lack of experience with pegvaliase during pregnancy. Pregnancy testing before initiating pegvaliase therapy is also required in some clinic protocols.

5.2 Consider continuing pegvaliase during pregnancy, weighing the risks of the known teratogenic effects of high blood PHE on the fetus with the unknown risks of pegvaliase, and incorporating the woman's preferences.

It is well established that elevated blood PHE is teratogenic to a fetus. The Maternal Phenylketonuria Collaborative Study reported outcomes of 468 pregnancies and 331 live births in pregnant women with PKU and demonstrated that uncontrolled blood PHE concentrations above 600 µmol/L were associated with an increased risk for miscarriage, major birth defects (including microcephaly and major cardiac malformations), intrauterine fetal growth retardation, and future intellectual disability with low IQ [21, 36].

In one case-study, a 22-year-old woman with PKU had been maintained on pegvaliase (10 mg/day) for 7 months when her pregnancy was identified. She discontinued pegvaliase for one week until a discussion with the metabolic team resulted in resuming pegvaliase therapy at the same dose. The decision was made based on her history of high blood PHE on diet therapy alone. During the pregnancy, there were several dose adjustments based on low blood PHE and/or concern about potential hypoPHE. While the birth outcome was normal, her pregnancy was associated with anxiety due to a psychological aversion to high protein foods, as well as logistical issues, including sporadic blood PHE monitoring, dietary PHE records, and time lags between the medical team's decision to adjust the dose of pegvaliase and the availability of the new dose [37].

Three pregnancies in two women with PKU managed on pegvaliase were reported at one metabolic clinic. The first pregnancy came to attention 6 weeks after the woman initiated pegvaliase therapy. The individual presented to the emergency department with abdominal pain and was found to be having a miscarriage. She continued pegvaliase therapy and at 19 weeks of therapy had a second pregnancy. She elected to discontinue pegvaliase, but miscarried the pregnancy late in the first trimester. The second woman became pregnant while on pegvaliase and decided to remain on therapy because her blood PHE was in good control. She had an early pregnancy loss at 7 weeks gestation during her 50th week of pegvaliase therapy [18].

Considering the known risk of MPKU, and in accordance with published guidance on pegvaliase use, pegvaliase use during pregnancy should be considered on a case-by-case basis [8]. Clinic protocols reviewed stated that if pregnancy occurs, the clinic should be contacted immediately and enrollment into the maternal pegvaliase surveillance program is recommended [21].

5.3 If a woman continues pegvaliase during pregnancy, aim for blood PHE concentrations of 120–360 µmol/L and monitor blood PHE weekly to bi-weekly.

There is currently insufficient experience in pregnant women treated with pegvaliase to recommend a blood PHE treatment range that is different from the 120–360 µmol/L in published guidelines for PKU [2]. While some clinics advocate for tighter blood PHE control during pregnancy, there is concern about low blood PHE in pregnancy impacting fetal growth, and pegvaliase therapy may increase the possibility of hypoPHE. In one case study of pegvaliase continued during pregnancy, low blood PHE was concerning and challenging to manage [37].

Consensus concerning optimal blood PHE during pregnancy while on pegvaliase therapy was not reached; respondents commented about the concern of low blood PHE as well as the "safe" upper limit for blood PHE in MPKU.

5.4 If a woman receiving pegvaliase therapy is considering pregnancy and chooses to discontinue pegvaliase and resume a PHE-restricted diet, she should discontinue pegvaliase at least 4 weeks prior to a planned pregnancy.

This recommendation aligns with published guidelines based on pharmacokinetic data showing a 4-week washout period is needed to clear pegvaliase. This guidance also suggested that pegvaliase therapy not be recommended for women of childbearing age who plan to become pregnant within 1 year [8].

However, in a 2021 GMDI poster presentation by Kopesky and Sperl, researchers suggested that being of childbearing age should not be a deterrent to offering pegvaliase, as it is possible for women to successfully return to a PHE-restricted diet prior to conception. The authors reported successful transition from pegvaliase to a PHE-restricted diet in two women, and described four pregnancies resulting in three live births without complications and one stillbirth. It was hypothesized that improved blood PHE control while receiving pegvaliase may improve executive function and help women achieve better adherence when returning to a PHE-restricted diet. One of the women described in the poster was off pegvaliase for 7 months prior to her first pregnancy and remained off for 5 years (with 3 pregnancies during this time), while another woman discontinued pegvaliase at nearly 6 weeks gestation. Both women were able to maintain good blood PHE control during pregnancy with diet alone and then resume pegvaliase therapy, reaching efficacy and consuming a normal protein intake without medical food [38].

5.5 For women who are returning to a PHE-restricted diet for pregnancy, frequent nutrition counseling and support is necessary.

The PKU diet is difficult to follow in adulthood [39] and resuming a PHE-restricted diet after consuming a more liberal diet is even more difficult. Reinstating medical food is also challenging. In one report the transition from discontinuation of pegvaliase to re-initiating a diet restriction appropriate for pregnancy occurred over 8–10 weeks and was successful, but required close nutrition counseling and support to maintain blood PHE between 120 and 360 µmol/L [40].

5.6 For a woman who wishes to breast-feed her infant, consider pegvaliase therapy during lactation with frequent monitoring of maternal blood PHE.

There are no published studies in humans regarding use of pegvaliase during lactation. In one study in rats, pegvaliase was detected in rat milk but not in the pups, and pegvaliase administration at high doses during lactation resulted in decreased pup weight and survival. The prescribing information for pegvaliase states that pegvaliase may cause low PHE content of human milk, but no data are available [21].

Pegvaliase is unlikely to be absorbed from breastmilk as pegvaliase is a polypeptide that is likely destroyed by proteases in the infant's gastrointestinal tract [41]. In addition, pegvaliase actively metabolizes only the free amino acid form of PHE and the PHE intake in a breastfed infant is bound in intact protein (mainly lactalbumin). Therefore, any trace amount of undetected PAL activity in breast milk will not alter infant nutrition [40]. The pegvaliase prescribing information recommends careful monitoring of blood PHE concentrations in breast-feeding women to prevent hypoPHE [21].

One case report describes a woman with PKU who discontinued pegvaliase during pregnancy and lactation. The case highlighted the difficulty of maintaining a PHE-restricted diet after childbirth. This report also included the analysis of one sample of pumped breast milk, taken after the woman resumed pegvaliase (20 mg/day). Comparisons with a control breast milk sample from a woman without PKU and a laboratory standard showed the activity of PAL in the affected woman's sample was indistinguishable from the comparison samples. The authors concluded that resuming pegvaliase during lactation would be considered in future pregnancies [40]. In the only reported case study of a woman on pegvaliase during pregnancy, the individual chose not to breast-feed her infant [37]. Women choosing to remain off pegvaliase should be counseled regarding necessary dietary management during lactation.

In the Delphi 2 survey, there was consensus for considering pegvaliase use during lactation.

5.7 Consider pegvaliase for adolescents (≥ 16 years of age) with PKU, taking into consideration level of blood PHE control, neurocognitive status, and quality of life on current therapy, as well as the individual’s goals for therapy.

No data are available for pegvaliase therapy in individuals under the age of 16 years. There is limited evidence for adolescents from the early phases of the clinical trials where individuals over 16–18 years were enrolled [39]. In subsequent trials, participants were 18 years or older. Of the 261 individuals in Study 301, only 11 were between 16 and 18 years of age at enrollment. All 11 individuals had inadequate blood PHE control (> 600 µmol/L) at baseline. These individuals received the same induction/titration/maintenance regimen (with a maximum dose of 40 mg/day) as those at least 18 years of age. Of the 11 individuals initially enrolled in the study, timing of response to pegvaliase and AEs reported were similar in type and frequency to the adult study population [42]. In Europe, pegvaliase is approved in doses up to 60 mg/d for individuals 16 years of age or older who have inadequate blood PHE control on prior management [17, 42]. In adolescents who are still growing, attention should be paid to avoid any possible effects of hypoPHE blood PHE levels.

A recent case series reporting on several individuals on pegvaliase therapy while in college has implications for adolescents. Authors noted that it is important to consider the individual's life events when initiating pegvaliase, as too much flux can make it difficult to manage induction and titration and may lead to treatment discontinuation. One individual discontinued pegvaliase as she feared significant side effects while away from home [20].

There was unanimous agreement in Delphi survey responses that adolescents at least 16 years of age should be offered pegvaliase if blood PHE is > 600 µmol/L.

## Discussion and conclusions

Pegvaliase therapy allows the possibility for traditional PKU dietary therapy to be discontinued, while still maintaining the benefits of blood PHE control. This necessitates a significant change in nutrition recommendations and education to support optimal nutritional status. The purpose of this update to the web-based GMDI/SERN PKU Nutrition Management Guideline is to provide new guidance for clinical use of pegvaliase. Recommendations focus on nutrition management and on the critical need for individualized, intensive nutrition counseling to achieve a healthy diet for all individuals with PKU receiving pegvaliase therapy. Recommendations are also focused on the opportunities and challenges experienced by individuals with PKU as a result of therapy and diet changes. This update provides a new paradigm in the transition of care practices for both clinicians and individuals with PKU.

The development of recommendations relied heavily on published US and European guidance regarding the use of pegvaliase. Recommendations regarding initiating therapy and managing response to therapy were corroborated by consensus among experts who have clinical experience with pegvaliase for the treatment of PKU.

These updated recommendations for nutrition management with pegvaliase therapy support consistency of best practice and provide guidance for changes in management perspective required, both by clinicians and by individuals with PKU. Whether individuals receiving successful pegvaliase therapy have followed recommended dietary restrictions into adulthood, or have chosen to discontinue them, new education and counseling is essential. Individuals with a history of treatment adherence have been dependent on medical foods to provide sufficient protein, on dietary restrictions to manage blood PHE levels, and on foods naturally low in protein and specialty low protein foods to provide adequate energy intake and food quantity. Successful pegvaliase therapy allows these individuals to safely consume more natural and higher protein foods. Benefits of successful pegvaliase therapy include improved neurocognition and psychosocial well-being associated with blood PHE control, improved nutritional status resulting from a healthy unrestricted diet, and improved quality of life with diet liberalization. Nutritional management does not become unnecessary, but rather requires a shift in perspective, understanding, and goals. Clinicians must provide education and counsel on how to achieve this more normal diet within a healthy nutrition context.

Similarly, for those individuals who have not been successful in adhering to dietary therapy, education and counseling is needed to ensure more healthy food choices. Experience with supporting the nutritional status of individuals with PKU who transition to an unrestricted diet containing more intact protein and discontinuation of medical food, is limited. Ongoing concerns of nutrition management should include those of nutrient adequacy as well as overnutrition associated with an unrestricted diet.

Changing lifetime eating patterns is difficult. This is especially true for individuals who have been taught their mental development and health depend on following restrictions, or whose cognition has been impaired by lack of blood PHE control. It is essential to understand an individual’s limitations and anxieties, as well as their expectations and goals for therapy outcome. Best care is achieved when treatment choices, counseling, and provision of services take each individual into account.

Like all guidelines, these guidelines are meant to be followed using the provider's clinical judgement and considering the individual's specific circumstances.

## Limitations and future steps

These recommendations are derived from reported evidence from clinical trials, cross-sectional studies, case reports, and clinical practices reported in gray literature, as well as expert input utilizing Delphi surveys and Nominal Group processes. Because pegvaliase has only recently become commercially available, published experience with the nutrition management of this alternate therapy for PKU is limited, with reports from clinical trials forming the basis of knowledge. The strength of evidence for many of the recommendations is less than that for other clinical research publications, but is appropriate when compared to similar published guidelines. Strength of evidence for each recommendation may be found in Table 1.

An understanding of optimal care for individuals with PKU receiving pegvaliase therapy, and recognition of the need for change in practices for assessing and counseling individuals for transition to a healthy unrestricted diet, continue to evolve. Knowledge gaps in the literature and areas lacking consensus highlight the need for further study to increase the validity of these updated recommendations. A particular limitation is the paucity of experience reported for pegvaliase use in special populations, including pregnancy, lactation, adolescence, and childhood. There also is a need for effective assessment tools that can be used in the clinical setting, as well as resources for addressing food aversions and changing eating patterns in individuals receiving pegvaliase therapy.

The GMDI/SERN Nutrition Management Guidelines undergo periodic review and are updated based on the availability of new research and clinical advances. In order to maintain validity and effectiveness of these guidelines, ongoing research and clinical reports concerning new therapies such as pegvaliase, and how those therapies change nutrition management, is imperative.

## Data Availability

The datasets used and/or analysed during the current study are available from the corresponding author on reasonable request.

## Abbreviations

AE: Adverse event
ACMG: American College of Medical Genetics
BH4: Tetrahydrobiopterin
DRI: Dietary reference intakes
EMA: European Medicines Agency
FDA: Food and drug administration
FFQ: Food Frequency Questionnaire
GNO-METAB-L: Genetic nutrition online metabolic listserv
GMDI: Genetic metabolic dietitians international
hypoPHE: Hypophenylalaninemia
IMD: Inherited metabolic disorder
PAH: Phenylalanine hydroxylase
PAL: Phenylalanie ammonia lyase
PHE: Phenylalanine
PKU: Phenylketonuria
RD/RDN: Registered Dietitian/Registered Dietitian Nutritionist
REMS: Risk evaluation and mitigation strategies
SERN: Southeast regional genetics network
SIMD: Society for inherited metabolic disorders
TYR: Tyrosine
QoL: Quality of life
US: United States
